# DNA Damage, Genome Stability, and Adaptation: A Question of Chance or Necessity?

**DOI:** 10.3390/genes15040520

**Published:** 2024-04-21

**Authors:** John Herrick

**Affiliations:** Independent Researcher at 3, Rue des Jeûneurs, 75002 Paris, France; jhenryherrick@yahoo.fr

**Keywords:** non-adaptive radiation, mutation rates, species richness, species evenness, karyotype diversity, DNA repair systems

## Abstract

DNA damage causes the mutations that are the principal source of genetic variation. DNA damage detection and repair mechanisms therefore play a determining role in generating the genetic diversity on which natural selection acts. Speciation, it is commonly assumed, occurs at a rate set by the level of standing allelic diversity in a population. The process of speciation is driven by a combination of two evolutionary forces: genetic drift and ecological selection. Genetic drift takes place under the conditions of relaxed selection, and results in a balance between the rates of mutation and the rates of genetic substitution. These two processes, drift and selection, are necessarily mediated by a variety of mechanisms guaranteeing genome stability in any given species. One of the outstanding questions in evolutionary biology concerns the origin of the widely varying phylogenetic distribution of biodiversity across the Tree of Life and how the forces of drift and selection contribute to shaping that distribution. The following examines some of the molecular mechanisms underlying genome stability and the adaptive radiations that are associated with biodiversity and the widely varying species richness and evenness in the different eukaryotic lineages.

## 1. Introduction

### 1.1. Karyotype Diversity and Species Richness

The origins of the biodiversity comprising the Tree of Life involve longstanding and ongoing debates in evolutionary biology. Darwin characterized the astonishing species diversity among angiosperms (some 4000 species) as the “abominable mystery”: “the rapid development … of all higher order plants within recent geological times” [1]. The generation of biodiversity involves two fundamental but biologically independent variables: mutation and selection. Mutation events, which alter genotypes, have been assumed to occur randomly at the molecular and genetic levels, while natural selection is expected to act non-randomly on the correspondingly altered phenotypes at the individual and population levels.

A major question concerns how these two variables interact to establish an equilibrium, or balance, between the forces of mutation and selection during the process of speciation. Most mutations are deleterious to the organism and undergo negative, or purifying, selection, while other mutations are beneficial and undergo positive selection, or adaptation. A third class of mutation is neither beneficial nor harmful, but instead “neutral” or “nearly neutral”, meaning they are expected to have either negligible or no effect on an organism’s differential fitness. 

Neutral mutations become fixed, or substituted, in a population through random genetic drift rather than by Darwinian natural selection. The characteristic of being neutral has, along with other mutational events such as whole-genome amplifications (polyploidy), resulted in the astonishing range of genome sizes and architectures across the eukaryotic Tree of Life, in striking contrast to the relatively streamlined genome size range found in prokaryotes [2].

Eukaryotic genomes are organized in individual units (chromosomes) of differing numbers and sizes (karyotypes). The genomes themselves vary enormously in size across both plants and animals. Animal genome sizes, for example, range from 0.04 picograms (pg; C-value = IC) in *Trichoplax adhaerens* to 133 pg in *Protopterus aethiopicus*, or about 3300-fold. In plants, genome sizes range over 2400-fold from 0.06 pg to about 152 pg, the largest known eukaryote genome [3,4]. In contrast, the genomes of bacteria and Archaea range in size from 0.1 to 16 Mbp, or about 160-fold [5].

**Figure 1:**
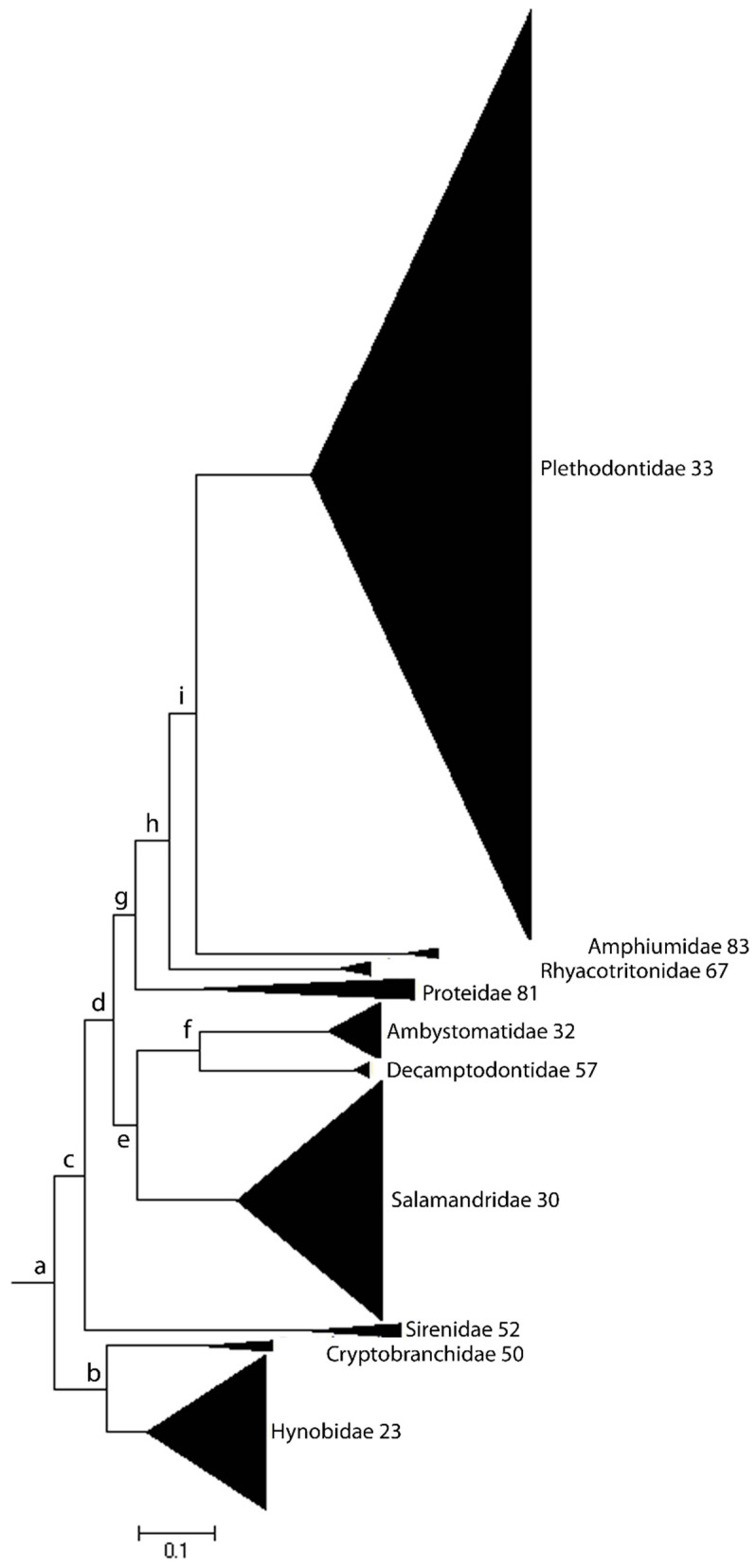
Species richness and evenness in Urodela. The black triangles represent species richness in the 10 salamander families. Species richness varies widely across the respective salamander lineages and their distributions are highly uneven. The numbers refer to family average genome size. Families with smaller average genome size (less than 40 pg) are more speciose than families with larger genome size (greater than 40 pg), independent of being sister clades (e.g., Hynobiidae–Cryptobranchidae and Amphiumidae–Plethodontidae).

Changes in karyotypes and genome sizes are closely associated with corresponding differences in species richness within different taxonomic groups, such as mammals and salamanders. Karyotype diversity (KD), moreover, is highly correlated with species richness (number of species in a clade) across closely and distantly related clades. The evenness in karyotype diversity also correlates with species evenness in the mammalian phylogenetic tree, evenness reflecting the extent of balance/imbalance in the distribution of either KD or species richness (SR). In each case, the respective size distributions are significantly skewed in parallel, with lower KD aligning with lower SR and vice-versa. Extreme differences in species richness and evenness in the Urodela phylogenic tree, for example, are readily apparent (Figure 1).

### 1.2. Framing the Question: What Is the Role of Genome Stability in Karyotype Evolution and Species Diversity?

A still unanswered question arises, at least concerning the mammalian lineage: are the correlations between KD and SR and the correlations of evenness in the SR and KD distributions a trivial consequence of Neo-Darwinian natural selection acting on random genome modifications and rearrangements? Or do the correlations reflect processes of non-adaptive radiation that result from a balance between mutational inputs and neutral substitutions in a population? In other words, is speciation initially an adaptively neutral phenomenon resulting from relaxed selection, which generates the genetic diversity on which Darwinian natural selection ultimately acts? And if so, to what extent can relaxed selection and genetic drift explain or account for the widely varying distributions of SR observed in most, if not all, animal and plant lineages?

The question is pertinent given that most of the eukaryotic genome comprises non-coding, apparently neutral DNA that derives principally from a variety of different transposable elements (TEs). This “neutral DNA” is largely responsible for the 64,000-fold range in genome sizes found in eukaryotes [6], yet the number of genes in any given lineage varies little. The average number of genes in vertebrates, for example, is about 20,000, while the average number in invertebrates is about 16,000 [7]. Rats and humans have approximately the same number of genes: 22,000 [8]. Although neutral, the location of TEs in the genome is not random but compartmentalized in the form of late replicating gene-poor heterochromatin.

It would appear then that species in a lineage differ from each other more in the amounts of non-coding DNA than in the amount of DNA on which natural selection is expected to act, namely genes, regulatory elements, and the organization of genes in the genome (synteny). Synteny in mammals, for example, has been remarkably conserved during the evolution of the lineage [9], yet the mammalian genome has a highly variable karyotype (2n = 6–7 in the Indian muntjac to over 100 in some rodents) and a significantly large range in C-value of about 358-fold [10,11].

**Figure 2:**
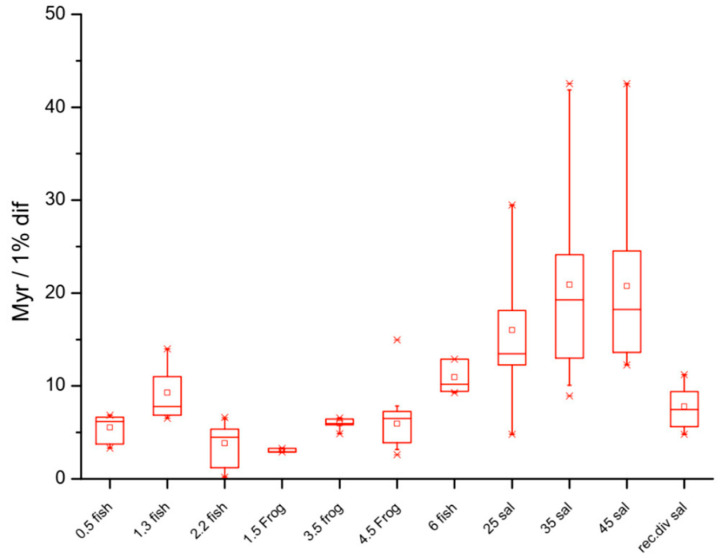
Salamanders have relatively slower rates of molecular evolution compared to frogs and fish. Units of evolutionary period (UEP: million years per 1% difference in the POMC gene in different lineages) reflect evolutionary rates. The box plots reveal an increase with the genome size, indicating that lineages associated with larger genomes have correspondingly slower rates of evolution. The box corresponds to the middle 50% of the data and the whiskers to 80%; the small square corresponds to the mean and the line to the median. Mean UEP values are significantly different (*p* < 0.05) for the pairs 1.3 to 2.2 pg (fish–fish), 4.5 to 6 pg (frog–fish), 4.5 to 35 pg (frog–salamander), and 6 to 35 pg (fish–salamander). Recently diverged salamanders (far right: rec div sal) appear to be evolving faster than other salamanders that diverged earlier; recently, diverged fish (2.2 fish, Salmonidae) are also evolving faster than other fish that diverged earlier. Note, however, that salamanders are evolving more slowly than Salmonidae, despite the lineages having diverged at about the same time. A clear trend of slower rates of evolution in older lineages is apparent in each group. Lineage-specific effects on evolutionary rates are also apparent independently of the genome size: Salmonidae (C-value 2.2 pg) are evolving at a faster rate than other fish lineages. Likewise, cartilaginous fish (*Heterodontus francisci*) and different members of the Actinoptergyii class (C-value between 5 and 7 pg) are evolving much more slowly than the other lineages. It should be noted that phylogenetic relatedness within each taxon is not specified. The plots therefore represent the non-phylogenetic relationship between the genome size and the divergence rate across the fish, anurans, and urodeles: genome size, independently of lineage, correlates with divergence rate when compared across the three different vertebrate groups.

Although the genome size often correlates negatively with the rates of evolution in plants and animals, genome stability and rates of change in C-value appear to underlie the rates of macroevolution [12]. Salamanders, for example, have large and highly conserved genomes that evolve very slowly (C-value: > 10 pg) and frogs have smaller genomes that have much faster rates of evolution (C-value: < 10 pg), while mammals have rates of genome evolution that are 20× faster than those of anurans and a much more restricted range of C-value compared to that of the Amphibia (Figure 2). Importantly, synteny is also highly conserved in frogs and salamanders [13].

Remarkably, natural selection has not purged the eukaryotic genome of this ostensibly useless DNA, suggesting that it might play a role in adaptation and speciation, for example, in consolidating reproductive isolation [14]. While non-coding DNA itself might be biologically inert, the heterochromatin that it forms plays a number of vital roles in transcription, DNA repair, DNA replication timing, and differentiation and development. The following looks at the potential biological functions of non-coding DNA and heterochromatin in relation to the factors contributing to adaptation and speciation.

## 2. Non-Adaptive Radiation: Ecological Selection vs. Genetic Drift

Motoo Kimura proposed a hypothesis of non-adaptive radiation (NAR) based on genetic drift, or the random fixation of an allele or genotype in a population [15]. The Neo-Darwinian hypothesis, in contrast, holds that natural selection acting on an advantageous, variant phenotype is the primary and principal driver behind fixing a genotype variant in a population [16,17]. The NAR hypothesis rests on the assumption that substitution rates equal mutation rates (mutation/substitution balance): mutation rates determine the rates of substitution and, consequently, the rates of speciation. 

Although genetic drift might drive a mutation to substitution and fixation in a group with a small effective population size (N*e*), Kimura’s NAR does not assume that Darwinian natural selection plays a minor or insignificant role in establishing reproductive isolation, for example, through the effects of speciation genes, genomic modifications resulting in incompatible karyotypes and other pre-and post-zygotic barriers to gene flow [18]. It remains unclear, however, how these two evolutionary forces, drift and selection, interact during the processes of speciation and adaptive radiation [19].

Theories of non-adaptive radiation have been proposed ever since Darwin. Non-adaptive radiation corresponds to lineage diversification in the absence of environmental shifts or evident niche divergence [20,21,22]. In contrast to ecological-based theories of non-adaptive radiations, Kimura’s theory focuses on niche neutral genotype radiations at the molecular genetic level. The theory rests on four fundamental stages defining the speciation process:

(1) Relaxation of a selective constraint (a weakened negative, or purifying, selection) resulting in a burst in the number of new gene and genotype variants;
(2) Differential fixation of variants in a population, or subpopulations, under the force of genetic drift;
(3) Rapid habitat-driven diversification into new niches and environments (ecological selection);
(4) Competitive exclusion between related groups leading to extensive adaptive evolution and radically different taxa following successful adaptation to new ecological niches.

A substantial amount of evidence has accumulated in support of the NAR hypothesis since it was first formulated in 1991. The role of relaxed selection in influencing evolutionary rates is well established in plants and animals (Stage 1) [23,24,25,26]. Relaxed purifying selection is associated with changes in the genome size (both expansions and contractions) and altered genome architecture and karyotypes [27,28]. The role of genetic drift in modulating genome sizes, however, remains unclear (Stage 2), but is expected to contribute significantly in the ancestral population during the early stages of adaptive radiation [29,30,31]. The expected increase in mutational loads under relaxed selection in isolated groups with small effective population size (N*e*) enhances the levels of standing genetic variation under the conditions of balancing selection (Stage 3) [32,33,34,35]. Balancing selection acts to maintain diversity in a population over long periods of time [36,37]. The corresponding elevated levels of genetic diversity (GD), in turn, promote speciation when variants invade new niches and habitats (Stage 4). Population differentiation, for example, is related to speciation rates over evolutionary time [38].

Implicit in the NAR hypothesis is a time lag between stage 1 (stochastic divergence between isolated populations) and stage 4 (ecological selection and adaptation) [39]: the four stages take place in succession, or nearly in succession, over millions of years rather than simultaneously or in parallel [40]. On a microevolutionary scale, diversification without morphological change has been observed in plants, lizards, and salamanders: the rates of species diversification are not coincident with ecological and phenotypic evolution, while ecological and phenotypic evolution co-occur in time as expected according to ecological speciation [41]. These findings are more consistent with a primarily niche neutral diversification model than with the models of simple density-dependent diversification [42]. Hence, the speciation process corresponds to a repeated cycle of niche neutral diversification followed by a period of density-dependent ecological adaptation. 

Other examples of neutral genotype diversification relate to genotype–phenotype maps and the neutral sets or networks they form [43]. More than one genotype can code for a single phenotype. The size distribution of neutral sets varies substantially, with any given phenotype mapping to multiple genotypes [44]. Since ecological selection acts on the individual phenotype, neutral sets of genotypes indicate a widely varying amount of degeneracy that is perhaps a signature of genetic drift [45]. 

Genotype–phenotype degeneracy can then be seen as analogous to the degeneracy in the genetic code [46,47], which provided an initial insight into the neutral theory of evolution. The neutral divergence of the genotype is therefore operating within the selective constraints that fix a phenotype in a population [48]. Phenotype plasticity and “epigenetic drift”, or the accumulation of stochastic epigenetic modifications, can also generate other forms of neutral and non-neutral genomic and genetic diversity [49,50,51,52]. Another example of protein evolution via the force of genetic drift concerns rapidly evolving intrinsically disordered proteins, which increase in number with organism complexity [53].

## 3. Genome Stability and Rates of Speciation: Karyotype Diversity versus Gene Diversity in Determining Species Richness

As early as the 1970s, a clear distinction had been established between karyotype diversity and genetic diversity and their respective relation to species richness [54,55]. Taken together, the observations suggested that “evolution at the organismal level is correlated more highly with karyotype evolution than with structural gene evolution” [56]. Moreover, it was found that the rates of karyotype evolution varied significantly among different taxonomic groups, whereas the rates of change in structural genes were about the same. 

Another study found a negative correlation between the levels of gene heterozygosity and the rates of chromosomal speciation, suggesting that the rates of speciation increase in populations with a small effective population size (low heterozygosity) [57]. The only feasible way, however, of estimating the effective population size is to rely on measures of within-population nucleotide diversity at neutral genomic sites, such as silent sites in codons (dS) [58]. While the dependence of heterozygosity on the effective population size is necessarily true for isolated populations of the same species (same mutation rate per individual), it is not entirely clear whether the use of such measures can be applied to whole taxonomic groups for comparative studies [59]. Absolute rates of silent site divergence, for example, are 7× faster in angiosperms compared to gymnosperms [60], which might (or might not) affect biological conclusions based strictly on measures of effective population size.

Consistent with the earlier studies in the 1970s, other biological features such as genome stability also seem to be highly associated with evolutionary rates. The rates of genome evolution appear to be closely correlated with the levels of species richness. In mammals, a strong correlation between species richness and karyotype diversity was first reported in 1980. The author proposed that: “properties of stable or unstable karyotype may indicate that the cytological factors of importance are all of a submicroscopic nature” [61].

Among the three taxa referred to above, Urodela have the fewest number of species (816 newts and salamanders; time of emergence: 230 Mya) [62]. Anurans are substantially more speciose than salamanders (7682 frogs and toads; emerging 180 Mya) [63], and Mammalia have a similar number of species (6495, of which about one-third, or 2276, belong to Rodentia). Mammals first evolved 225 Mya, but experienced a rapid adaptive radiation 65.8 Mya among placentals, much later than the anuran radiation [64]. Hence, salamanders are evolving more slowly than frogs, which are evolving more slowly than mammals [65,66].

The question emerges from these and other observations: What are the submicroscopic factors that might explain the correlations between SR and KD and the manifest differences in SR and species evenness in the respective phylogenetic trees—assuming that those cellular and presumably nuclear factors and mechanisms are genuinely associated with the correlations and their respective differences? If that assumption holds true, to what extent then would those yet unidentified factors contribute to—or contrast with—the prevailing view that most, if not all, speciation events and adaptive radiations are attributable to ecological speciation alone, instead of to NARs resulting from DNA damage, mutation, and diversification? [16,67]. What these submicroscopic factors might be remains unknown.

## 4. DNA Damage Detection and Repair Systems (DDR) and Chromatin Structure

This section addresses the hypothesis mentioned above that the “properties of stable or unstable karyotype may indicate that the cytological factors of importance are *all* of a submicroscopic nature”, and therefore are crucially involved in driving the speciation events that determine species diversity. It should be emphasized that this hypothesis raises the question of how mutation rates interact with and mediate the forces of genetic drift and ecological selection during the process of speciation. 

What then might be the submicroscopic factors mentioned in the hypothesis? Kimura’s molecular-based NAR would seem to imply that they must be factors that mediate the balance between mutation and substitution during periods of both drift and selection. The DNA damage detection and repair systems in all organisms in the Tree of Life determine the balance between mutation input through DNA damage and misrepair and substitution output through drift and/or selection. Because the repair systems govern genome stability, they directly influence how karyotypes—including cancer karyotypes—evolve as a consequence of DNA damage and errors in DNA repair (mutations).

As the genome size increases, so does the probability of DNA damage and mutation. Genomes with larger amounts of functional DNA (number of genes, regulatory sequences, etc.) are therefore expected to have lower mutation rates; yet, it is well known that larger genomes are indeed more prone to DNA damage and mutation. The apparent paradox can be resolved by noting that the eukaryote genome is compartmentalized into two broad and varying forms of chromatin: euchromatin (EC) and heterochromatin (HC) [68]. Euchromatin is characterized by large DNA loops that are more accessible to regulatory enzymes and are more rich in genes. Heterochromatin, facultative or constitutive [69], is more compact, has a much lower gene density, and is more refractory to enzymes involved in DNA and RNA metabolism (replication, transcription, and repair).

This spatial compartmentalization also imposes temporal compartmentalization according to a replication timing (RT) program [70]: EC replicates early during the S-phase of the cell cycle and HC replicates late. Late-replicating DNA protects the genome and cell against mitotic catastrophe, or premature entry into the mitotic phase, which would damage unreplicated gene-dense EC and cause apoptosis. Late-replicating DNA also serves as a substrate for the ATR/ATM checkpoint system that mediates DNA repair by inhibiting the activation of late and/or dormant DNA replication origins until the cell is ready to recover from DNA damage at mid- to late S-phase. The S-phase and G2/M-phase checkpoint proteins Chk1 and Chk2 govern these functions and organize a multi-factorial cell cycle replication timing program.

Importantly, this temporal compartmentalization corresponds to the differential deployment of the two main eukaryote DNA repair systems: error-free homologous recombination (HR), which operates more efficiently in the open euchromatin that replicates early in S-phase, and error-prone non-homologous end-joining (NHEJ), which operates throughout S-phase but dominates in the G2/M and G1 phases [71,72,73]. The ratio between HC and NHEJ decreases with the genome size across eukaryotes: species with larger genomes rely more heavily on NHEJ than do species with smaller genomes [74]. Consequently, they tend to have much larger introns and higher intron density [75,76].

## 5. Mutation–Substitution Balance and Replication Timing

Individual mutation events, although assumed to occur stochastically, are not randomly distributed across the genome [77]. Several studies have established that the rates of mutation depend highly on the replication timing (RT) of subregions of the eukaryote genome. The RT program therefore serves to limit mutation rates in gene-rich EC: mutation rates are significantly higher in late-replicating HC [78,79,80,81,82,83,84], suggesting that mutation rates in early- and late-replicating DNA are anti-correlated to a degree directly proportional to the quantity of late-replicating HC relative to early-replicating EC—a hypothesis that remains to be validated. Additionally, DNA damage-prone polymerases, the Y-family of translesion polymerases, also might account for the higher mutation rate in late S-phase. The elevated mutation rates in late-replicating DNA in yeast, for example, are suppressed when DNA translesion polymerases are rendered inactive [79,82].

Not surprisingly, the functional identity of genes—gene ontology—is also unevenly distributed across the genome. Essential housekeeping genes, required for the survival of all cells, are universally early-replicating, while adaptive genes, such as the olfactory complex, are generally late-replicating and located in or near heterochromatic domains [85,86]. Speciation genes tend to be non-essential in contrast to housekeeping genes [87], and therefore genes involved in ecological adaptation are under more relaxed selection pressure in the genome. Again, early-replicating genes, such as housekeeping genes associated with EC, have substantially lower rates of molecular divergence than late-replicating genes associated with HC and ecological speciation/adaptation.

The late-replicating status of non-essential, mutation-prone speciation/adaptation genes remains to be firmly established, but some studies strongly suggest that the epigenome biases mutation rates [88,89], which might promote the adaptive functions exhibited by immune system genes, which are late-replicating, and other ecologically responsive genes, such as the rapidly evolving olfactory gene complex. The enhancement in genetic diversity in late-replicating genes—those associated with mutation-prone heterochromatin—can thus be viewed as analogous to the programed DNA-damaging processes involved in the generation of antibody diversity in the immune system [90]. 

It would not be far-fetched then to suggest, according to the analogy, that the location and organization of genes in the genome have evolved in order to promote the genetic diversity underlying species diversity and to limit, at the same time, deleterious mutations from occurring in early-replicating, essential genes. The lower deleterious mutation rates in essential genes would provide a selective advantage to the cell, while the higher rates of mutation in non-essential genes would provide an adaptive advantage to the organism. This analogy is all the more reasonable given that the rates of speciation (and therefore, the survival of a lineage) depend critically on the intra-population level of allele diversity, which is generated by the DDR system. 

While the analogy draws an admittedly anecdotal comparison between the generation of antibody diversity by clonal selection and the generation of genetic diversity by the DDR-HC complex, it nevertheless remains a fact that innate immune system genes and genes associated with adaptation mainly reside in or near late-replicating regions of mutation-prone heterochromatin and appear to experience relatively higher divergence rates. Essential early-replicating genes, in contrast, appear to be under a much more stringent selection pressure [88,89].

## 6. Life History Traits and the DDR System: Limb Regeneration and Maximum Lifespan

Species with large C-values have longer introns and correspondingly slower rates of transcription, a phenomenon known as “intron delay” [91,92]. Consequently, they have much slower cell and life cycles. Other features associated with species with either large or more stable genomes are long maximum lifespans (MLSs), slow rates of development, and in some cases the ability to regenerate ablated tissue [93]. Salamanders, for example, can regenerate not only limbs but also internal organs, including the brain [94]. Tissue regeneration depends on a strong DNA damage response system that converges on the cell cycle checkpoint regulators Chk1 and Chk2: inhibiting Chk1 and Chk2 impairs regeneration [95].

Given the role of the heterochromatin–DDR complex in RT, DNA repair, cell cycle regulation, limb regeneration, and aging, it is not surprising that an embryonic state of chromatin (which is heterochromatic) also facilitates the experimental cloning of animals [96]. This might suggest that the limb regeneration and slow aging phenotypes in salamanders are associated with the substantially larger amounts of heterochromatin in their genomes compared to those of other species with smaller genomes. At the taxonomic family level in the salamander lineage, obligate neotenes consistently have genomes much larger on average than metamorphic or direct developing salamanders [97,98]. Larger amounts of heterochromatin might therefore facilitate DNA repair, slow the rate of aging, enhance MLSs, and retard developmental rate. 

DNA and histone methylation are features of heterochromatin and are associated with developmental genes, gene regulatory regions and the polycomb-repressive complex 2, a histone methyl transferase associated with repressed, transcriptionally silent facultative heterochromatin, and X-chromosme inactivation [14]. Histone methylation also participates in the DDR system [99], indicating a direct mechanistic relationship between HC and DNA repair. Additionally, epigenetic drift involves the erosion of CpG methylation and is closely associated with aging: higher densities of CpG methylation buffer against epigenetic drift and extend MLSs [51]. Other important chromatin modulators, such as Sirt6, are also involved in the HC-DDR complex and influence mutation rates and aging [100]. The link between heterochromatin, genome stability, and aging perhaps can be extended to the rates of speciation [101,102,103].

## 7. Life History Traits and the DDR System: Cellular Differentiation and Development

When DNA damage occurs, cells face three possible outcomes depending on the amount of damage: (1) checkpoint-mediated cell cycle arrest and DNA repair (DDR activation), (2) cellular senescence (aging), and (3) apoptosis (programed cell death). A fourth fate involves cellular differentiation [104,105]. Apoptosis is an integral feature of both the DDR system and the cellular differentiation that drives embryogenesis and development [106]. Chk1 is activated, for example, at the midblastula transition during embryogenesis when the cellular transcription program is switched on [107]; and it acts to extend the cell cycle and initiate cellularization in the developing embryo [108].

It has been claimed that cellular differentiation, a feature driving the evolution of metazoans, emerged as a defense against lethal DNA damage and oncogenesis [105]. The idea is that cellular differentiation is an evolutionary adaptation to DNA damage and a prophylaxis against oncogenesis in metazoans. This raises an interesting question: are the rates of evolution constrained by the rates of development? The rates of development in salamanders, for example, are constrained by a nucleotypic effect relating to the genome size [109]. If so, could speciation rates scale with the timing of the program of differentiation and development in the individual organism? 

Additionally, the lower levels of DNA damage and the stronger DDR systems in euchromatin are expected consequences of an extended RT program that is due to higher levels of late-replicating heterochromatin. A prolonged RT program enhancing the DDR system might contribute to the slower rates of evolution observed in Urodela compared to Anura and Mammalia. Such a relationship is also apparent within the Urodela lineage: species richness at the family-level taxonomic clade is negatively correlated with the average C-value (Figure 1). Although the latter observation remains to be rigorously established, slow aging and longer developmental programs, which result in longer MLSs, provide the organism with more time to repair DNA damage, thus promoting the efficiency of DNA repair and enhancing genome stability by reducing mutation/substitution rates. 

**Figure 3:**
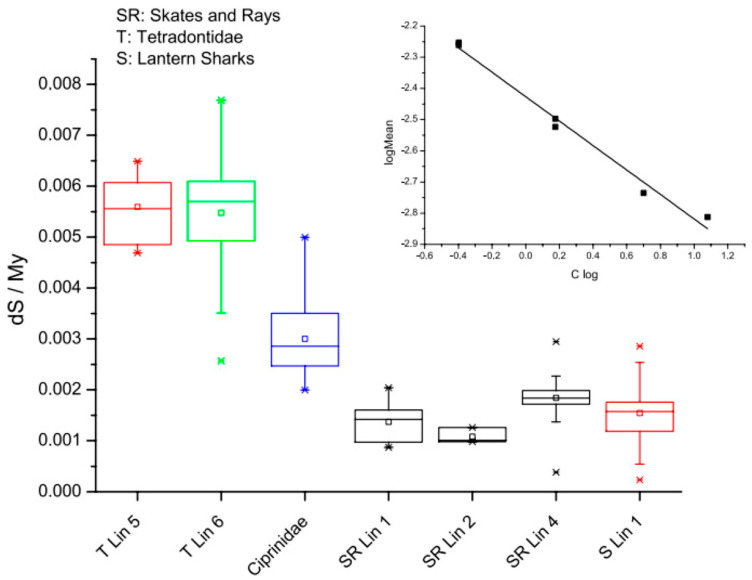
Rates of molecular evolution in the fish lineage. The box plots represent silent site substitutions in codons per million years (ds/Myr). Genetic distances were obtained from aligned sequences, and divergence times ascertained from the literature. Analyses were performed on Tetraodontiformes (T) (average C-value: 0.5 pg), Cypriniformes (1.5 pg), skates and rays (SR) (4 pg), and lantern sharks (S) (12 pg). A clear difference in evolutionary rates associated with the genome size is apparent. Note that skates, rays, and sharks all have exceptionally low and similar evolutionary rates. Inset: log-transformed data indicate a power law relationship between evolutionary rates and the genome size across these samples. The exponent is −0.39, suggesting significantly different modes of evolution in fish with small genomes compared to fish with larger genomes, perhaps because of the slower rates of DNA loss in species with larger genomes and a corresponding differential dependence on DNA repair systems between species with large versus small genomes (small C-value: HR > NHEJ; large C-value: HR < NHEJ). See [115].

If mutation rates set rates of speciation as Kimura’s NAR hypothesis proposes and if mutation rates vary substantially across animal and plant lineages, the DDR system and HC must play important roles in determining mutation and speciation rates across the Tree of Life (Figure 3). Speciation rates might indeed be related to developmental rates [110], a question that has long intrigued evolutionary and developmental biologists. This would suggest that, in more than just a metaphorical sense (though not exactly in a literal sense), “ontogeny recapitulates phylogeny” [111,112]. It would be interesting, nonetheless, to investigate how the rates of speciation and phylogenesis scale with the rates of development and ontogenesis, should it turn out that the DDR system and heterochromatin are, in fact, limiting for cell cycle progression and mutation [113,114,115,116].

## 8. Life History Traits and the DDR system: Sharks, Salamanders, and Resistance to Genetic Diseases

Selection and drift also operate at the organism level in the generation of a variety of gene-related diseases [117]. Genetic drift resulting from small or reduced effective population sizes is expected to result in relaxed selection on DNA repair systems according to what has been called the “Drift Barrier Hypothesis” [58]. As the breeding population size decreases, selection pressure on the efficiency and effectiveness of DNA repair systems will correspondingly decreases. Mutation and substitution rates will subsequently increase, and consequently the genome sizes will tend to increase through the accumulation of mutagenic TEs [26]. The result will necessarily be an increase in genetic diseases, for example, age-related diseases and cancer. 

Genetic drift can also result in somatic genome mosaicism, which has been associated with a variety of genetic diseases, including autism spectrum disorders and Alzheimer’s disease [117]. In the case of cancer, mutations can undergo somatic evolution and become clonally amplified either by genetic drift or because of a selective growth advantage that allows the cancer cell to develop into a tumor. Long-lived, slowly evolving species. such as sharks, salamanders, and other long-living species, all have very stable genomes and karyotypes, and are all highly resistant to oncogenesis. It is becoming increasingly clear that, in cases of a greater genome stability, resistance to cancer and longer maximum lifespans can be attributed to more efficient and effective DNA repair systems, in particular to a greater reliance on the fast-acting NHEJ DNA repair pathway as the genome size expands.

## 9. Discussion

This review has attempted to adumbrate some of the various mechanisms in which heterochromatin and DNA repair might play a role in maintaining genome integrity and stability, biological features that are increasingly associated with the rates of speciation and adaptive radiations [118]. The central question addressed in this paper concerns to what extent the molecular mechanisms mediating genome dynamics determine the rates of evolution in parallel to, or even in concert with, gene-specific mutation rates.

Mutation rates in vertebrates, for example, are very similar to the rates of TE transposition [119,120,121], which is regulated by heterochromatin and ecological variables that shape phenotype plasticity. A role for TE activity in punctuated equilibria has also been suggested [122]. Heterochromatin, however, might not be in and of itself a determining factor of SR and KD, but instead might operate more indirectly through the multiple pathways, both molecular and ecological, that affect and influence evolutionary outcomes. It has now become clear, however, that heterochromatin plays vital regulatory roles in RT, DNA repair, transcription, and development. Its role in speciation merits further investigation.

Alternatively, the evolution of chromatin, meaning the histone code, itself might play a decisive role in speciation not only by reorganizing the transcriptome within and across species but also by controlling the differential reliance on DNA repair pathways when the genome size expands or contracts: larger, more stable genomes rely predominantly on error-prone NHEJ repair [123]. The choice between repair pathways depends on post-translational histone marks, such as acetylation, methylation, ubiquitination, etc. [124]. In yeast and metazoa, the DNA replication factor Rif1 governs RT and determines the choice between DNA repair pathways: specifically, Rif1 blocks HR and promotes NHEJ. A highly abundant histone mark, H4K20me2, present on nucleosomes in G1 and G2 phases of the cell cycle, promotes the preferential use of NHEJ repair [73,125]. The evolution of the RT program and its coordination with the DDR system and chromatin status (constitutive HC, facultative HC, and EC), therefore, differentially integrate DNA and RNA metabolism in a manner unique to each karyotype and each species. 

## 10. Does Evolution Proceed by Repeated Cycles of Genetic Drift and Ecological Speciation?

**Figure 4:**
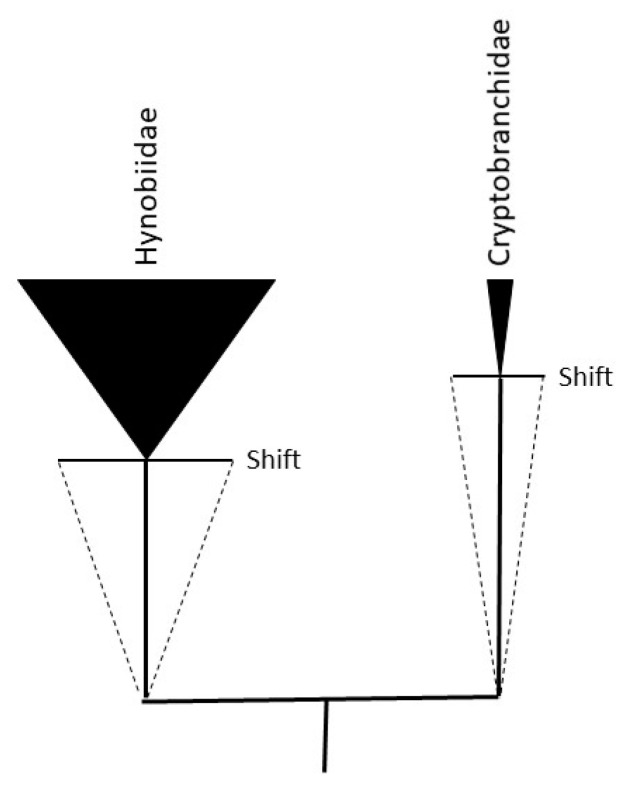
Hypothetical biphasic model of stem and crown group dynamics. The black triangles represent extant crown group family species richness (salamanders). The dotted triangles represent extinct stem group species richness. The Cryptobranchidae, for example, are evolving more slowly over evolutionary time (slope of dotted line; stem to crown age) than the sister clade of Hynobiidae. It is assumed in this case that speciation occurs in a predominantly neutral niche mode (neutral adaptive radiation) in the ancestral population until an environmental crisis, or shift, drives ecological speciation (adaptive radiation). Both drift and selection, however, are expected to shape simultaneously evolutionary paths. If karyotype diversity evolves neutrally (genetic drift) during an ancestral phase of evolution (dotted triangles), the rate of karyotype diversification might be greater than or at least equal to the rate of genetic diversification (rate KD ≥ rate GD): more than one genotype, for example, specifying a single phenotype. If an environmental shift applies selection pressure on the diversified karyotypes, a transition might take place where selection pressure acts principally, but not exclusively, on genes (rate of GD > rate of KD). The figure depicts one of multiple cycles generating extant species richness during the evolution of a lineage (crown group). The ancestral karyotypes surviving the post-crisis/shift will contribute proportionally to the karyotype diversity in the crown group until those ancestors become extinct (Gause’s principle). It should be noted that “living fossils”—the notion that stem group species persist into extant crown group species—is not assumed in this model: the rates of molecular evolution over time (molecular clock) will result in crown group species that are descended—and genetically distinct—from extinct stem group species, even in the absence of identifiable morphological or phenotypic change. Figure adapted from [127].

NAR, in its molecular formulation, might imply a biphasic mode of evolution: (1) a lag period of drift involving chromosomal and genome rearrangements in a neutral niche occupied by an ancestor population (stem group), followed by (2) niche diversification and Neo-Darwinian positive selection on adaptive genes resulting in ecological speciation (crown group) [126,127] (Figure 4). The fact that synteny is highly conserved in salamanders, frogs, and mammals, while the rates of change in structural genes are fairly constant, supports the proposal that karyotypes evolve neutrally, whereas the transcriptome and its corresponding phenotypes evolve according to positive (and purifying) selection. It is also notable that the amount of synteny conservation is correlated with MLSs in mammals (unpublished).

Both features, karyotype diversification and genetic diversification, might contribute successively or in tandem (and in concert) to the processes of reproductive isolation and adaptation. Might there then be two distinct molecular clocks determining the mode and tempo of evolution: a gene-based molecular clock that sets a constant rate of genetic evolution across lineages and a genome/junk-based molecular clock that sets a given rate of speciation that varies from lineage to lineage? The amount of junk DNA in salamander lineages, for example, increases linearly with the phylogenetic age [128,129]. 

The central tenet of Kimura’s NAR hypothesis relies on the assumption that mutation rates directly influence substitution rates (mutation–substitution balance), and therefore, speciation rates. Ecological speciation, in contrast, rests on the assumption that environmental shifts acting on functional DNA alone (or predominantly) determine speciation rates. It has been repeatedly found in every organism examined to date (including salamanders) that substitutions at non-silent sites in gene codons (amino acid substitutions) are correlated with substitutions at silent sites, suggesting that selection acts not only on genes but also on gene locations and regions in the genome (e.g., early- vs. late-replicating DNA and heterochromatin vs. euchromatin) [88].

This raises an interesting, perhaps provocative, question: To what extent do mutation rates and DNA repair efficiencies influence, or set, substitution rates—and hence speciation rates—independently of ecological selection? It has been pointed out that “locational selection would have to be realized through the influence of the local mutation rate on the amino acid changing mutation rate” [88]. If this hypothesis is correct—selection based on gene location—and if it is a reflection of the non-random distribution of DNA damage/mutation events, it would not be unreasonable to expect that such a relationship/correlation among gene location, DNA damage, and DNA repair efficiency would apply not only within genomes, but also across taxa (salamanders vs. frogs vs. mammals) in a manner that sets variations in speciation rates within lineages and explains, at least in part, the striking differences in species richness and evenness observed in the Tree of Life.

## 11. Conclusions

What role, if any, non-coding DNA and heterochromatin play in the process of speciation and adaptive radiations remains largely unknown and awaits further investigation into the finer molecular details of genome architecture and DNA metabolism. The growing body of genome-wide data from different taxa and the emergence of new bioinformatic tools have opened up the field of the genomics of speciation by allowing detailed analyses at the levels of the gene, the genome, and the transcriptome [130,131,132].

Other lines of investigation will further integrate cell cycle regulation and DNA metabolism into the existing and future theoretical models, clarifying the role of DNA metabolism in the process of speciation that is, to date, incompletely understood. In short, the molecular basis of NAR suggests that the evolutionary outcomes of totipotent cells forming different lineages within a single metazoan organism during development, and the evolutionary outcomes of an individual organism/population forming a new species within a genus during speciation, might be as much a question of chance and genetic drift as it is a question of necessity and ecological adaptation.

## References

[1] Soltis P.S., Folk R.A., Soltis D.E. (2019). Darwin review: Angiosperm phylogeny and evolutionary radiations. Proc. R. Soc. B Biol. Sci..

[2] Lynch M. (2006). Streamlining and Simplification of Microbial Genome Architecture. Annu. Rev. Microbiol..

[3] Pellicer J., Hidalgo O., Dodsworth S., Leitch I.J. (2018). Genome Size Diversity and Its Impact on the Evolution of Land Plants. Genes.

[4] Pellicer J., Fay M.F., Leitch I.J. (2010). The largest eukaryotic genome of them all?. Bot. J. Linn. Soc..

[5] Rodríguez-Gijón A., Buck M., Andersson A.F., Izabel-Shen D., A Nascimento F.J., Garcia S.L. (2023). Linking prokaryotic genome size variation to metabolic potential and environment. ISME Commun..

[6] Hidalgo O., Pellicer J., Christenhusz M., Schneider A., RLeitch A.R., Leitch I.J. (2017). Is There an Upper Limit to Genome Size?. Trends Plant Sci..

[7] Prachumwat A., Li W.-H. (2008). Gene number expansion and contraction in vertebrate genomes with respect to invertebrate genomes. Genome Res..

[8] Demuth J.P., De Bie T., Stajich J.E., Cristianini N., Hahn M.W. (2006). The Evolution of Mammalian Gene Families. PLoS ONE.

[9] Damas J., Corbo M., Kim J., Turner-Maier J., Farré M., Larkin D.M., Ryder O.A., Steiner C., Houck M.L., Hall S. (2022). Evolution of the ancestral mammalian karyotype and syntenic regions. Proc. Natl. Acad. Sci. USA.

[10] Graphodatsky A.S., A Trifonov V., Stanyon R. (2011). The genome diversity and karyotype evolution of mammals. Mol. Cytogenet..

[11] Gregory T.R. (2005). The C-value Enigma in Plants and Animals: A Review of Parallels and an Appeal for Partnership. Ann. Bot..

[12] Puttick M.N., Clark J., Donoghue P.C. (2015). Size is not everything: Rates of genome size evolution, not *C*-value, correlate with speciation in angiosperms. Proc. R. Soc. B Biol. Sci..

[13] Voss S.R., Kump D.K., Putta S., Pauly N., Reynolds A., Henry R.J., Basa S., Walker J.A., Smith J.J. (2011). Origin of amphibian and avian chromosomes by fission, fusion, and retention of ancestral chromosomes. Genome Res..

[14] Liu J., Ali M., Zhou Q. (2020). Establishment and evolution of heterochromatin. Ann. N. Y. Acad. Sci..

[15] Kimura M. (1991). The neutral theory of molecular evolution: A review of recent evidence. Jpn. J. Genet..

[16] Schluter D., Conte G.L. (2009). Genetics and ecological speciation. Proc. Natl. Acad. Sci. USA.

[17] Ravinet M., Faria R., Butlin R.K., Galindo J., Bierne N., Rafajlović M., Noor M.A.F., Mehlig B., Westram A.M. (2017). Interpreting the genomic landscape of speciation: A road map for finding barriers to gene flow. J. Evol. Biol..

[18] Martin C.H., Richards E.J. (2019). The Paradox Behind the Pattern of Rapid Adaptive Radiation: How Can the Speciation Process Sustain Itself Through an Early Burst?. Annu. Rev. Ecol. Evol. Syst..

[19] Gillespie R.G., Bennett G.M., De Meester L., Feder J.L., Fleischer R.C., Harmon L.J., Hendry A.P., Knope M.L., Mallet J., Martin C. (2020). Comparing Adaptive Radiations Across Space, Time, and Taxa. J. Hered..

[20] Czekanski-Moir J.E., Rundell R.J. (2019). The Ecology of Nonecological Speciation and Nonadaptive Radiations. Trends Ecol. Evol..

[21] Schenk J.J. (2021). The Next Generation of Adaptive Radiation Studies in Plants. Int. J. Plant Sci..

[22] Kozak K.H., Weisrock D.W., Larson A. (2006). Rapid lineage accumulation in a non-adaptive radiation: Phylogenetic analysis of diversification rates in eastern North American woodland salamanders (Plethodontidae: *Plethodon*). Proc. R. Soc. B Biol. Sci..

[23] Wertheim J.O., Murrell B., Smith M.D., Pond S.L.K., Scheffler K. (2015). RELAX: Detecting Relaxed Selection in a Phylogenetic Framework. Mol. Biol. Evol..

[24] Hunt B.G., Ometto L., Wurm Y., Shoemaker D., Yi S.V., Keller L., Goodisman M.A.D. (2011). Relaxed selection is a precursor to the evolution of phenotypic plasticity. Proc. Natl. Acad. Sci. USA.

[25] Persi E., Wolf Y.I., Koonin E.V. (2016). Positive and strongly relaxed purifying selection drive the evolution of repeats in proteins. Nat. Commun..

[26] Lynch M., Conery J.S. (2003). The Origins of Genome Complexity. Science.

[27] Fuselli S., Greco S., Biello R., Palmitessa S., Lago M., Meneghetti C., McDougall C., Trucchi E., Stabelli O.R., Biscotti A.M. (2023). Relaxation of Natural Selection in the Evolution of the Giant Lungfish Genomes. Mol. Biol. Evol..

[28] Mohlhenrich E.R., Mueller R.L. (2016). Genetic drift and mutational hazard in the evolution of salamander genomic gigantism. Evolution.

[29] Ai B., Wang Z.-S., Ge S. (2012). Genome size is not correlated with effective population size in the *oryzas* pecies. Evolution.

[30] Whitney K.D., Baack E.J., Hamrick J.L., Godt M.J.W., Barringer B.C., Bennett M.D., Eckert C.G., Goodwillie C., Kalisz S., Leitch I.J. (2010). A role for nonadaptive processes in plant genome size evolution?. Evolution.

[31] Blommaert J. (2020). Genome size evolution: Towards new model systems for old questions. Proc. R. Soc. B Biol. Sci..

[32] Bourgeois Y., Boissinot S. (2019). On the Population Dynamics of Junk: A Review on the Population Genomics of Transposable Elements. Genes.

[33] Llaurens V., Whibley A., Joron M. (2017). Genetic architecture and balancing selection: The life and death of differentiated variants. Mol. Ecol..

[34] Ding G., Hasselmann M., Huang J., Roberts J., Oldroyd B.P., Gloag R. (2021). Global allele polymorphism indicates a high rate of allele genesis at a locus under balancing selection. Heredity.

[35] Avise J.C. (1977). Genic heterozygosity and rate of speciation. Paleobiology.

[36] Charlesworth D. (2006). Balancing Selection and Its Effects on Sequences in Nearby Genome Regions. PLoS Genet..

[37] Bitarello B.D., Brandt D.Y.C., Meyer D., Andrés A.M. (2023). Inferring Balancing Selection From Genome-Scale Data. Genome Biol. Evol..

[38] Harvey M.G., Seeholzer G.F., Smith B.T., Rabosky D.L., Cuervo A.M., Brumfield R.T. (2017). Positive association between population genetic differentiation and speciation rates in New World birds. Proc. Natl. Acad. Sci. USA.

[39] Mani G.S., Clarke B.C. (1990). Mutational order: A major stochastic process in evolution. Proc. R. Soc. London. Ser. B. Biol. Sci..

[40] Uyeda J.C., Hansen T.F., Arnold S.J., Pienaar J. (2011). The million-year wait for macroevolutionary bursts. Proc. Natl. Acad. Sci. USA.

[41] Folk R.A., Stubbs R.L., Mort M.E., Cellinese N., Allen J.M., Soltis P.S., Soltis D.E., Guralnick R.P. (2019). Rates of niche and phenotype evolution lag behind diversification in a temperate radiation. Proc. Natl. Acad. Sci. USA.

[42] Aguilée R., Gascuel F., Lambert A., Ferriere R. (2018). Clade diversification dynamics and the biotic and abiotic controls of speciation and extinction rates. Nat. Commun..

[43] Rezazadegan R., Reidys C. (2018). Degeneracy and genetic assimilation in RNA evolution. BMC Bioinform..

[44] Mohanty V., Greenbury S.F., Sarkany T., Narayanan S., Dingle K., Ahnert S.E., Louis A.A. (2023). Maximum mutational robustness in genotype—Phenotype maps follows a self-similar blancmange-like curve. J. R. Soc. Interface.

[45] Manrubia S., Cuesta J.A., Aguirre J., Ahnert S.E., Altenberg L., Cano A.V., Catalán P., Diaz-Uriarte R., Elena S.F., García-Martín J.A. (2021). From genotypes to organisms: State-of-the-art and perspectives of a cornerstone in evolutionary dynamics. Phys. Life Rev..

[46] Whitacre J.M., Atamas S.P. (2012). Degeneracy allows for both apparent homogeneity and diversification in populations. Biosystems.

[47] Paaby A.B., Rockman M.V. (2014). Cryptic genetic variation: Evolution’s hidden substrate. Nat. Rev. Genet..

[48] Wideman J.G., Novick A., A Muñoz-Gómez S., Doolittle W.F. (2019). Neutral evolution of cellular phenotypes. Curr. Opin. Genet. Dev..

[49] Maleszka R., Mason P.H., Barron A.B. (2014). Epigenomics and the concept of degeneracy in biological systems. Briefings Funct. Genom..

[50] Milosavljevic A. (2011). Emerging patterns of epigenomic variation. Trends Genet..

[51] Bertucci-Richter E.M., Parrott B.B. (2023). The rate of epigenetic drift scales with maximum lifespan across mammals. Nat. Commun..

[52] Charlesworth D., Barton N.H., Charlesworth B. (2017). The sources of adaptive variation. Proc. R. Soc. B Biol. Sci..

[53] Afanasyeva A., Bockwoldt M., Cooney C.R., Heiland I., Gossmann T.I. (2018). Human long intrinsically disordered protein regions are frequent targets of positive selection. Genome Res..

[54] Wilson A.C., Sarich V.M., Maxson L.R. (1974). The Importance of Gene Rearrangement in Evolution: Evidence from Studies on Rates of Chromosomal, Protein, and Anatomical Evolution. Proc. Natl. Acad. Sci. USA.

[55] Bush G.L., Case S.M., Wilson A.C., Patton J.L. (1977). Rapid speciation and chromosomal evolution in mammals. Proc. Natl. Acad. Sci. USA.

[56] Maxson L.E.R., Wilson A.C. (1979). Rates of molecular and chromosomal evolution in salamanders. Evolution.

[57] Coyne J.A. (1984). Correlation between Heterozygosity and Rate of Chromosome Evolution in Animals. Am. Nat..

[58] Lynch M., Ali F., Lin T., Wang Y., Ni J., Long H. (2023). The divergence of mutation rates and spectra across the Tree of Life. Embo Rep..

[59] Roddy A.B., Alvarez-Ponce D., Roy S.W. (2021). Mammals with Small Populations Do Not Exhibit Larger Genomes. Mol. Biol. Evol..

[60] De La Torre A.R., Li Z., Van de Peer Y., Ingvarsson P.K. (2017). Contrasting Rates of Molecular Evolution and Patterns of Selection among Gymnosperms and Flowering Plants. Mol. Biol. Evol..

[61] Bengtsson B.O. (1980). Rates of karyotype evolution in placental mammals. Hereditas.

[62] Schoch R.R., Werneburg R., Voigt S. (2020). A Triassic stem-salamander from Kyrgyzstan and the origin of salamanders. Proc. Natl. Acad. Sci. USA.

[63] Portik D.M., Streicher J.W., Wiens J.J. (2023). Frog phylogeny: A time-calibrated, species-level tree based on hundreds of loci and 5242 species. Mol. Phylogenet. Evol..

[64] Hunter P. (2020). The rise of the mammals: Fossil discoveries combined with dating advances give insight into the great mammal expansion. EMBO Rep..

[65] Bredeson J.V., Mudd A.B., Medina-Ruiz S., Mitros T., Smith O.K., Miller K.E., Lyons J.B., Batra S.S., Park J., Berkoff K.C. (2024). Conserved chromatin and repetitive patterns reveal slow genome evolution in frogs. Nat. Commun..

[66] Liedtke H.C., Gower D.J., Wilkinson M., Gomez-Mestre I. (2018). Macroevolutionary shift in the size of amphibian genomes and the role of life history and climate. Nat. Ecol. Evol..

[67] Rundell R.J., Price T.D. (2009). Adaptive radiation, nonadaptive radiation, ecological speciation and nonecological speciation. Trends Ecol. Evol..

[68] Janssen A., Colmenares S.U., Karpen G.H. (2018). Heterochromatin: Guardian of the Genome. Annu. Rev. Cell Dev. Biol..

[69] Zylicz J.J., Heard E. (2020). Molecular Mechanisms of Facultative Heterochromatin Formation: An X-Chromosome Perspective. Annu. Rev. Biochem..

[70] Nakatani T., Schauer T., Altamirano-Pacheco L., Klein K.N., Ettinger A., Pal M., Gilbert D.M., Torres-Padilla M.-E. (2024). Emergence of replication timing during early mammalian development. Nature.

[71] Mao Z., Bozzella M., Seluanov A., Gorbunova V. (2008). DNA repair by nonhomologous end joining and homologous recombination during cell cycle in human cells. Cell Cycle.

[72] Mao Z., Bozzella M., Seluanov A., Gorbunova V. (2008). Comparison of nonhomologous end joining and homologous recombination in human cells. DNA Repair.

[73] Chen Z., Tyler J.K. (2022). The Chromatin Landscape Channels DNA Double-Strand Breaks to Distinct Repair Pathways. Front. Cell Dev. Biol..

[74] Sonoda E., Hochegger H., Saberi A., Taniguchi Y., Takeda S. (2006). Differential usage of non-homologous end-joining and homologous recombination in double strand break repair. DNA Repair.

[75] Vinogradov A.E. (1999). Intron—Genome Size Relationship on a Large Evolutionary Scale. J. Mol. Evol..

[76] Farlow A., Meduri E., Schlötterer C. (2011). DNA double-strand break repair and the evolution of intron density. Trends Genet..

[77] Kin C., Dmitry A. (2015). Gordenin Clusters of Multiple Mutations: Incidence and Molecular Mechanisms. Annu. Rev. Genet..

[78] Stamatoyannopoulos J.A., Adzhubei I., Thurman R.E., Kryukov G.V., Mirkin S.M., Sunyaev S.R. (2009). Human mutation rate associated with DNA replication timing. Nat. Genet..

[79] Lang G.I., Murray A.W. (2011). Mutation Rates across Budding Yeast Chromosome VI Are Correlated with Replication Timing. Genome Biol. Evol..

[80] Pink C.J., Hurst L.D. (2009). Timing of Replication Is a Determinant of Neutral Substitution Rates but Does Not Explain Slow Y Chromosome Evolution in Rodents. Mol. Biol. Evol..

[81] Weber C.C., Pink C.J., Hurst L.D. (2012). Late-Replicating Domains Have Higher Divergence and Diversity in Drosophila melanogaster. Mol. Biol. Evol..

[82] Agier N., Fischer G. (2012). The Mutational Profile of the Yeast Genome Is Shaped by Replication. Mol. Biol. Evol..

[83] Chen C.-L., Rappailles A., Duquenne L., Huvet M., Guilbaud G., Farinelli L., Audit B., D’Aubenton-Carafa Y., Arneodo A., Hyrien O. (2010). Impact of replication timing on non-CpG and CpG substitution rates in mammalian genomes. Genome Res..

[84] Murat P., Perez C., Crisp A., van Eijk P., Reed S.H., Guilbaud G., Sale J.E. (2022). DNA replication initiation shapes the mutational landscape and expression of the human genome. Sci. Adv..

[85] Bomblies K., Peichel C.L. (2022). Genetics of adaptation. Proc. Natl. Acad. Sci. USA.

[86] Bracci A.N., Dallmann A., Ding Q., Hubisz M.J., Caballero M., Koren A. (2023). The evolution of the human DNA replication timing program. Proc. Natl. Acad. Sci. USA.

[87] Wu C.-I., Ting C.-T. (2004). Genes and speciation. Nat. Rev. Genet..

[88] Chuang J.H., Li H. (2004). Functional Bias and Spatial Organization of Genes in Mutational Hot and Cold Regions in the Human Genome. PLoS Biol..

[89] Monroe J.G., Srikant T., Carbonell-Bejerano P., Becker C., Lensink M., Exposito-Alonso M., Klein M., Hildebrandt J., Neumann M., Kliebenstein D. (2022). Mutation bias reflects natural selection in Arabidopsis thaliana. Nature.

[90] Cohn M., Mitchison N.A., Paul W.E., Silverstein A.M., Talmage D.W., Weigert M. (2007). Reflections on the clonal-selection theory. Nat. Rev. Immunol..

[91] Heyn P., Kalinka A.T., Tomancak P., Neugebauer K.M. (2015). Introns and gene expression: Cellular constraints, transcriptional regulation, and evolutionary consequences. BioEssays.

[92] Chakra M.A., Isserlin R., Tran T.N., Bader G.D. (2021). Control of tissue development and cell diversity by cell cycle-dependent transcriptional filtering. eLife.

[93] Sessions S.K., Wake D.B. (2021). Forever young: Linking regeneration and genome size in salamanders. Dev. Dyn..

[94] Joven A., Elewa A., Simon A. (2019). Model systems for regeneration: Salamanders. Development.

[95] Sousounis K., Bryant D.M., Fernandez J.M., Eddy S.S., Tsai S.L., Gundberg G.C., Han J., Courtemanche K., Levin M., Whited J.L. (2020). Eya2 promotes cell cycle progression by regulating DNA damage response during vertebrate limb regeneration. eLife.

[96] Lemaitre J.-M., Danis E., Pasero P., Vassetzky Y., Méchali M. (2005). Mitotic Remodeling of the Replicon and Chromosome Structure. Cell.

[97] Gregory T.R. (2002). Genome size and developmental complexity. Genetica.

[98] Mueller R.L., E Cressler C., Schwartz R.S., A Chong R., A Butler M. (2023). Metamorphosis Imposes Variable Constraints on Genome Expansion through Effects on Development. Integr. Org. Biol..

[99] Gong F., Miller K.M. (2019). Histone methylation and the DNA damage response. Mutat. Res. Mol. Mech. Mutagen..

[100] Tian X., Firsanov D., Zhang Z., Cheng Y., Luo L., Tombline G., Tan R., Simon M., Henderson S., Steffan J. (2019). SIRT6 Is Responsible for More Efficient DNA Double-Strand Break Repair in Long-Lived Species. Cell.

[101] Crofts S.J.C., Latorre-Crespo E., Chandra T. (2024). DNA methylation rates scale with maximum lifespan across mammals. Nat. Aging.

[102] Pértille F., Da Silva V.H., Johansson A.M., Lindström T., Wright D., Coutinho L.L., Jensen P., Guerrero-Bosagna C. (2019). Mutation dynamics of CpG dinucleotides during a recent event of vertebrate diversification. Epigenetics.

[103] Venney C.J., Anastasiadi D., Wellenreuther M., Bernatchez L. (2023). The Evolutionary Complexities of DNA Methylation in Animals: From Plasticity to Genetic Evolution. Genome Biol. Evol..

[104] Sjakste N., Riekstiņa U. (2021). DNA damage and repair in differentiation of stem cells and cells of connective cell lineages: A trigger or a complication?. Eur. J. Histochem..

[105] Sherman M.H., Bassing C.H., Teitell M.A. (2011). Regulation of cell differentiation by the DNA damage response. Trends Cell Biol..

[106] Meier P., Finch A., Evan G. (2000). Apoptosis in development. Nature.

[107] Zhang M., Kothari P., Mullins M., Lampson M.A. (2014). Regulation of zygotic genome activation and DNA damage checkpoint acquisition at the mid-blastula transition. Cell Cycle.

[108] Farrell J.A., Shermoen A.W., Yuan K., O’Farrell P.H. (2012). Embryonic onset of late replication requires Cdc25 down-regulation. Genes Dev..

[109] Jockusch E.L., Kruuk L.E.B., Gilchrist J.S. (1997). An evolutionary correlate of genome size change in plethodontid salamanders. Proc. R. Soc. B Biol. Sci..

[110] Jablonski D. (2020). Developmental bias, macroevolution, and the fossil record. Evol. Dev..

[111] Arthur W. (2002). The emerging conceptual framework of evolutionary developmental biology. Nature.

[112] Uesaka M., Kuratani S., Irie N. (2022). The developmental hourglass model and recapitulation: An attempt to integrate the two models. J. Exp. Zool. Part B Mol. Dev. Evol..

[113] Brownstein C.D., MacGuigan D.J., Kim D., Orr O., Yang L., David S.R., Kreiser B., Near T.J. (2024). The genomic signatures of evolutionary stasis. Evolution.

[114] Herrick J. (2011). Genetic variation and dna replication timing, or why is there late replicating dna?. Evolution.

[115] Sclavi B., Herrick J. Slow Evolution of *rag1* and *pomc* Genes in Vertebrates with Large Genomes. Submitted on 9 February 2013. https://arxiv.org/ftp/arxiv/papers/1302/1302.2182.pdf.

[116] Sendell-Price A.T., Tulenko F.J., Pettersson M., Kang D., Montandon M., Winkler S., Kulb K., Naylor G.P., Phillippy A., Fedrigo O. (2023). Low mutation rate in epaulette sharks is consistent with a slow rate of evolution in sharks. Nat. Commun..

[117] Vijg J. (2021). From DNA damage to mutations: All roads lead to aging. Ageing Res. Rev..

[118] Jagannathan M., Yamashita Y.M. (2021). Defective Satellite DNA Clustering into Chromocenters Underlies Hybrid Incompatibility in *Drosophila*. Mol. Biol. Evol..

[119] Ricci M., Peona V., Guichard E., Taccioli C., Boattini A. (2018). Transposable Elements Activity is Positively Related to Rate of Speciation in Mammals. J. Mol. Evol..

[120] Quadrana L., Etcheverry M., Gilly A., Caillieux E., Madoui M.-A., Guy J., Silveira A.B., Engelen S., Baillet V., Wincker P. (2019). Transposition favors the generation of large effect mutations that may facilitate rapid adaption. Nat. Commun..

[121] Baduel P., Quadrana L., Hunter B., Bomblies K., Colot V. (2019). Relaxed purifying selection in autopolyploids drives transposable element over-accumulation which provides variants for local adaptation. Nat. Commun..

[122] Zeh D.W., Zeh J.A., Ishida Y. (2009). Transposable elements and an epigenetic basis for punctuated equilibria. BioEssays.

[123] Hughes S.E., Hawley R.S. (2009). Heterochromatin: A Rapidly Evolving Species Barrier. PLOS Biol..

[124] Clouaire T., Legube G. (2015). DNA double strand break repair pathway choice: A chromatin based decision?. Nucleus.

[125] Krenning L., Berg J.v.D., Medema R.H. (2019). Life or Death after a Break: What Determines the Choice?. Mol. Cell.

[126] Halliday T.J.D., dos Reis M., Tamuri A.U., Ferguson-Gow H., Yang Z., Goswami A. (2019). Rapid morphological evolution in placental mammals post-dates the origin of the crown group. Proc. R. Soc. B Biol. Sci..

[127] Budd G.E., Mann R.P. (2020). The dynamics of stem and crown groups. Sci. Adv..

[128] Kumar S. (2005). Molecular clocks: Four decades of evolution. Nat. Rev. Genet..

[129] Martin C.C., Gordon R. (1995). Differentiation trees, a junk DNA molecular clock, and the evolution of neoteny in salamanders. J. Evol. Biol..

[130] Bock D.G., Cai Z., Elphinstone C., González-Segovia E., Hirabayashi K., Huang K., Keais G.L., Kim A., Owens G.L., Rieseberg L.H. (2023). Genomics of plant speciation. Plant Commun..

[131] Wolfsberger W.W., Battistuzzi F.U., Oleksyk T.K. (2022). Genomics of Adaptation and Speciation. Genes.

[132] Campbell C.R., Poelstra J.W., Yoder A.D. (2018). What is Speciation Genomics? The roles of ecology, gene flow, and genomic architecture in the formation of species. Biol. J. Linn. Soc..

